# The impact of an educational program on recognition, treatment and report of child abuse

**DOI:** 10.1186/s13052-017-0389-1

**Published:** 2017-08-14

**Authors:** Pietro Ferrara, Antonio Gatto, Nunzia Pia Manganelli, Francesca Ianniello, Maria Elisa Amodeo, Maria Amato, Ida Giardino, Antonio Chiaretti

**Affiliations:** 1 Institute of Pediatrics, Catholic University Medical School, Rome, Italy; 2 Service of Pediatrics, Campus Bio-Medico University, Rome, Italy; 3 Department of Clinical and Experimental Medicine, University of Foggia, Foggia, Italy

**Keywords:** Child abuse, Maltreatment, Neglect, Training, Pediatricians

## Abstract

**Background:**

Pediatricians play a crucial role in the identification and management of child abuse and neglect (CAN) but they often don’t have a formal specialized training.

**Methods:**

We analysed retrospectively data about patients, 0 - 18 years of age, victims of CAN between 1 April 2005 and 30 April 2015. The aim of the study was to evaluate the effect of a multidisciplinary educational program, “CAN: prevention strategies, individuation and treatment”, on the knowledge, case recognition, treatment and follow-up of physicians of Gemelli University Hospital in Rome, regarding physical, sexual abuse and neglect. This program, in 3 different editions biannually, respectively in May-July 2010, November-January 2012 and February-May 2014, was based on 4 sessions, each one of 2 days.

**Results:**

Considering the number of victims of CAN between 2005 and 2015 we observed 66 cases of maltreatment. We divided the study population in 2 groups: group A, before the educational programs, patients evaluated from 1 April 2005 to 30 July 2010; group B, after the educational program from 1 August 2010 to 30 April 2015. We observed 23 children in group A and 43 children in group B with an improvement of 87%. Analyzing our data about sex, nationality, type of perpetrators, we found that: 37/66 (56%) of children were females compared to 29/66 (44%) males; 41/66 (62%) of children came from Italy compared to 25/66 (38%) of foreign children; 52/66 (79%) of the perpetrators of abuse were parents or family members compared to acquaintances 10/66 (15%) and to strangers 4/66 (6%).

**Conclusions:**

Considering the prevalence of CAN, the need to develop clinically competent clinicians and the improving of residency education in child maltreatment is imperative. Improving the clinical skills of pediatricians to identify and evaluate CAN may lead to reduce morbidity and mortality of these children.

## Background

Pediatricians play a crucial role in the identification and management of child abuse and neglect (CAN). They are the main investigator of recognizing and reporting suspected CAN. All physicians, in particular pediatricians, in CAN often are called on by courts as expert witnesses. Actually many physicians, however, have no formal specialized training in CAN. In 2008, a review of the professional education landscape found that no specific residency accreditation requirement exists for training in CAN [1].

Although there have been recent increases in recognition of CAN as a medical problem, there is no correlation of improved knowledge among physicians. Many pediatric residents leave residency with limited clinical training in CAN. After graduation, the programs of didactic training on CAN are described as very useful by physicians, but these didactic training are not mandatory so only a low number of physicians complete these elective activities.

Some authors demonstrated that the diagnosis of CAN is improved when physicians are adequately trained to recognize inflicted injuries and medical signs of abuse [2, 3]. Future research on training in CAN management and report is needed because there is very scarce and low-quality evidence on the accuracy of instruments for identifying abused children. Child maltreatment is mostly identified when children have already serious consequences and the sensitivities and specificities of tools are inadequate [4]. Before considering a screening program of child maltreatment, better knowledge on the beginning of child maltreatment and development of valid screening instruments at subclinical stages remain necessary [4].

## Methods

We analysed retrospectively data about patients, 0 - 18 years of age, evaluated at Gemelli University Hospital in Rome, victims of CAN between 1 April 2005 and 30 April 2015.

The aim of the study was to evaluate the effect of a multidisciplinary educational program, “CAN: prevention strategies, individuation and treatment”, on the knowledge, case recognition, treatment and follow-up of physicians of Gemelli University Hospital regarding physical, sexual abuse and neglect. This program, in 3 different editions biannually, respectively in May-July 2010, November-January 2012-2013 and February-May 2014, was based on 4 sessions, each one of 2 days. The 1st session based on a general overview of CAN, the 2nd on physical abuse, the 3rd on sexual abuse and 4th on neglect and legal considerations; each of the 4 sessions was designed to consider epidemiology, interviewing of victims, physical evaluation, behavioural, physical indicators and legal sequellae.

Using the centralized database of the Emergency Department (ED), accessed via GIPSE software, we identified the patients’ number evaluated in ED and discharged with diagnosis of “child maltreatment or child sexual abuse”.

To obtain the number of all patients who were both victims of CAN and hospitalized, but who were not diagnosed with CAN after the ED department evaluation, we analysed the archives of Gemelli Hospital Social Service. We consulted the medical record of each patient searching for data about age, sex, nationality, anamnesis, physical examination and diagnostic examinations.

In the absence of gold standard for ensuring identification of CAN, we condidered some proposed standards such us the number of evaluations at ED for each patient, after or before the hospitalization during the study period, expert assessments, substantiation by the child protection services or other social services, diagnosis by a medical, social or judicial team using one or several information sources (caregivers or child interview, child symptoms, child physical examination, and other medical record review).

We divided the study population in 2 groups: group A, before the educational programs, patients evaluated from 1 April 2005 to 30 July 2010; group B, after the educational program from 1 August 2010 to 30 April 2015.

We analysed data about categories of maltreatment (sexual abuse, physical abuse and neglect), age, gender, nationality. We also evaluated different type of aggressors, time of visits, injury severity and hospital admission. We highlighted also children victims of abuse with complex needs or difficult social conditions or with a risk patterns (living in foster care, drug-addicted-parents).

This is a retrospective study, designed to analyse pre-existing data, collected from the medical records of patients, therefore there are some limitations such as selection bias and misclassification or information bias.

The study was carried out in compliance with the Helsinki Declaration and was approved by the Institute of Pediatrics of Catholic University, Rome.

## Results

In ED 19 patients between 2005 and 2015 were evaluated with diagnosis of CAN. Four children (group A) received diagnosis between 1 April 2005 and 30 July 2010 (2 physical abuses, 2 sexual abuses); 15 (group B) between 1 August 2010 and 30 April 2015 (4 physical abuses, 11 sexual abuses) with an improvement of maltreatment diagnosis over 300%. Sixty-three percent of patients (12/19) were female, 37% (7/19) male; 32% (6/19) were victims of physical abuse, 68% (13/19) were victims of sexual abuse (2 cases of oral sex, 2 of sexual penetration, 9 of sexual violence without penetration). The most common injury identified in these patients during the ED visit was ecchymosis with only one case of bite marks.

Evaluating the patients by age we observed that the mean age of patients victims of physical abuse was 6.0 years old and patients victims of sexual abuse was 11.6 years old. The perpetrators of abuse were parents or family members (13/19 - 68%), acquaintances (out of family) (4/19 - 21%) and stranger (2/19 - 11%).

Considering nationality, 13 (68%) children came from Italy compared to 6 (32%) foreign children (usually from South East Europe). After the evaluation at ED, 23% (3/13) sexually abused patients were discharged and 77% (10/13 patients) were hospitalized. Between the patients evaluated at ED 3/19 required a Neuropsychiatric treatment for the onset of panic attack, anxiety and sleep disorders.

Considering the time of admission to ED: 6/19 (32%) patients were evaluated from 8.00 am to 02.00 pm, 5/19 (26%) from 2.00 pm to 08.00 pm and 8/19 (42%) from 8.00 pm to 02.00 am.

Analysing the archives of Hospital Social Service, to estimate the number of all patients victims of CAN and hospitalized with a different diagnosis from child abuse after the evaluation in ED department, we found 47 cases. We identified this group of patients through the analysis of the medical record of each patient searching for data about anamnesis, physical examination and diagnostic examinations. Nineteen patients (group A), 28 patients (group B) with an increase of CAN diagnosis of about 50%. The diagnosis were physical maltreatment for 21 (45%) patients, (group A 10 cases, group B 11 cases), sexual abuse for 5 (10%) patients (group A 1 case, group B 4 cases), neglect for 21 (45%) patients, (group A 8 cases, group B 13 cases).

Evaluating the patients by sex and nationality we found that 53% (25/47) were females, 47% (22/47) males and 60% (28/47) came from Italy compared to 40% (19/47) foreign children. Clinical characteristics of physical maltreatment were summarized in Table 1. Fifteen children (32%) required Neuropsychiatric treatment, in particular 2 cases due to suicide attempt and psychosis 1 case.

**Table 1:** Clinical characteristics of physical maltreatment

Diagnosis	Cases
Head injury	7
Skin lesions	5
Poisoning	4
Polytrauma	3
Others	2

The perpetrators of abuse included parents or family members (39/47 - 83%), acquaintances (out of family) (6/47 - 13%) and strangers (2/47 - 4%).

Between the several categories of neglect we observed medical neglect in 43% of patients.

Considering the number of victims of CAN between 2005 and 2015 we observed 66 cases of maltreatment: 23 children in group A and 43 children in group B with an improvement of 87%. Summarising our data about sex, nationality, type of perpetrators, we found that:

- 37/66 (56%) of children were females compared to 29/66 (44%) males;
- 41/66 (62%) of children came from Italy compared to 25/66 (38%) of foreign children;
- 52/66 (79%) of the perpetrators of abuse were parents or family members compared to acquaintances 10/66 (15%) and to strangers 4/66 (6%).

Finally, considering children victims of abuse, with complex needs or difficult social conditions or with a risk patterns, we identified: 2 children lived in foster care, 3 with drug-addicted-parents and 1 with post traumatic stress disorder (Table 2).

**Table 2:** Characteristics of patients

		Group A 2005-2010			Group B 2010-2015			Group B vs Group A		
		ED	Hospitalization	total	ED	Hospitalization	total	ED	Hospitalization	total
Patients n (%)		4 (17.4)	19 (82.6)	23	15 (34.9)	28 (65.1)	43	+11 (55.0)	+9 (45.0)	+20
Sex	Male n (%)	1 (11.1)	8 (89.9)	9	6 (30.0)	14 (70.0)	20	+5 (45.4)	+6 (56.4)	+11
	Female n (%)	3 (21.4)	11 (78.6)	14	9 (39.1)	14 (60.9)	23	+6 (66.7)	+3 (32.3)	+9
Nationality	Italian children n (%)	3 (20.0)	12 (80.0)	15	10 (38.5)	16 (61.5)	26	+7 (63.6)	+4 (36.4)	+11
	Foreign children n (%)	1 (12.5)	7 (87.5)	8	5 (29.4)	12 (70.6)	17	+4 (44.4)	+5 (55.6)	+9
Abuse	Physical abuse n (%)	2 (16.7)	10 (83.3)	12	4 (26.7)	11 (73.3)	15	+2 (66.7)	+1 (33.3)	+3
	Sexual abuse n (%)	2 (66.7)	1 (33.3)	3	11 (73.3)	4 (26.7)	15	+9 (75.0)	+3 (25.0)	+12
	Neglect n (%)	0 (0.0)	8 (100.0)	8	0 (0.0)	13 (100.0)	13	/	+5 (100.0)	+5

## Discussion

Our study demonstrated that training about CAN for pediatricians was associated with increased identification of victims of CAN.

The foundation of their competency in this role is established during their residency. Many authors have found areas of strength and weaknesses in current pediatric residency child abuse curricula [5, 6]. In the USA the recent creation of a Child Abuse Pediatrician subspecialty represents an opportunity to establish national standardized recommendations for child abuse training curricula and to identify several factors, including the presence of a written curriculum, that contribute to increased confidence among residents.

According to our study, available research demonstrates that different training strategies can improve knowledge and preparedness related to the recognition and response of CAN.

Starling et al. compared the training and knowledge in the United States among the 3 specialties most likely to encounter abused children: pediatrics, emergency medicine, and family medicine. Analyzing the survey received from 53 program directors and 462 residents they found that pediatric programs provide far more training and resources for child abuse education than emergency medicine and family medicine programs [5].

Narayan et al. analyzed with a survey the preparedness of residents to address CAN on graduation [7]. A 28-item survey was sent to chief residents of all 203 Accreditation Council for Graduate Medical Education–accredited pediatric residency programs in the United States from 2004 to 2005. The response rate was 71%. Respondents rated the levels of preparedness of graduating residents to address CAN as: very well (12%), well (54%), somewhat well (28%), or not well (6%). They demonstrated that the preparedness was significantly associated with hours of didactics, number of inpatient cases of CAN seen, number of sexual and physical abuse cases during mandatory rotation, and length of mandatory rotation [7]. They compared also their data with a similar study, by Dubowitz et al. conducted in 1988, and noted that not be a large difference between the perceived adequacy of training of residents over the two decades despite the impact of this problem on social and public health.

Hibbard et al. described the effect of a multidisciplinary project carried out in Indiana to educate medical, child protective and legal professional in the evaluation and care of children victims of child abuse [8]. The program was designed to accomplish 5 tasks: behavioural and physical indicator, interview, the use of dolls and drawing as interviewing aids, medical evaluation and legal responsibilities. The program was conducted with didactic presentation and discussions. They demonstrated an improved knowledge score at 2 weeks post-program and at 6 months [8].

In the literature, there is also a study by Showers et al. about the effect of self-instructional programs on physical and sexual abuse on emergency physicians [9]. The project was base on 2 self-instructional programs of about 6 h designed to study in the home or office. Topics in the didactic sections included incidence and consequences, legal aspects and documentation, risk and suspicion, types of abuse, interview techniques and treatment [9]. The project resulted in significant improvement in the knowledge.

These studies and experiences demonstrate that different tools can conduct to the same result and they are very useful to increase the best practice on this hot topic. In Italy actually there is no specific required training in CAN during graduation.

Our study suggests that the physicians should receive enough training to accurately identify and report abuse when it is suspected. Moreover, aside from identification, training can improve pediatricians’ ability to safely and sensitively respond to children exposed to maltreatment and their families, including improved communication in initial encounters, increased support to children and families in connecting with community services [10, 11]. First of all, is important to not underestimate symptoms such as abdominal pain and voiding disorders, in this cases it is fundamental to deal out organic causes for example taking drugs or rare diseases. So particularly, for abdominal pain and voiding disorders is important to rule out organic causes such as taking medication or other rare diseases [12].

Many programs should begin the process of implementing CAN training. In particular we believe that the CAN training not to be focused only on physical and sexual abuse but also on neglect or domestic violence. Neglect is the most common form of child abuse and the relationship between domestic violence and CAN has been well established [13–18].

Specific sessions on neglect and domestic violence training are mandatory in all didactic training programs on CAN.

There also is a need for a national CAN curriculum that can be adapted to any training site. Considering the incidence of CAN we think that the well-being of children depends on a well-trained and knowledgeable force of physicians who can identify and prevent CAN.

Characteristics of our sample may limit the generalization of the findings but our study is an example of efficacy of post-graduate training but to employ the medical practice on CAN, considering that child maltreatment is more prevalent than cancer and just as fatal, is necessary to have structured mandatory rotations for residencies. Furthermore the evolution of the concept of CAN leads to consider new types of maltreatment that in the future will certainly be taken into account with a new era of social paediatrics [19–21].

Domestic and interpersonal violence are public health problems so we think that it should warrant more attention during residency training on family violence as well as anatomy, molecular biology, genetic diseases or infective diseases.

## Conclusions

Considering the prevalence of CAN, the need to develop clinically competent clinicians and the improving of residency education in child maltreatment is imperative. Improving the clinical skills of pediatricians to identify and evaluate CAN may lead to reduce morbidity and mortality of these children. Training in early identification of CAN may improve outcomes of these children.

## Abbreviations

CAN: Child abuse and neglect
ED: Emergency Department
